# Soluble IgE‐binding factors in the serum of food‐allergic patients: Possible pathophysiological role of soluble FcεRI as protective factor

**DOI:** 10.1002/clt2.12222

**Published:** 2023-02-07

**Authors:** Carolin Steinert, Sherezade Moñino‐Romero, Monique Butze, Jörg Scheffel, Sabine Dölle‐Bierke, Josefine Dobbertin‐Welsch, Kirsten Beyer, Marcus Maurer, Sabine Altrichter

**Affiliations:** ^1^ Institute of Allergology Charité – Universitätsmedizin Berlin Freie Universität Berlin und Humboldt‐Universität zu Berlin Berlin Germany; ^2^ Allergology and Immunology Fraunhofer Institute for Translational Medicine and Pharmacology ITMP Berlin Germany; ^3^ Department of Biology, Chemistry and Pharmacy Freie Universität Berlin Berlin Germany; ^4^ University of Potsdam Potsdam Germany; ^5^ Division of Allergy and Immunology, Venerology and Allergy Department of Dermatology Charité – Universitätsmedizin Berlin Freie Universität Berlin und Humboldt‐Universität zu Berlin Berlin Germany; ^6^ Department of Pediatric Respiratory Medicine, Immunology and Critical Care Medicine Charité – Universitätsmedizin Berlin Freie Universität Berlin und Humboldt‐Universität zu Berlin Berlin Germany; ^7^ Department for Dermatology and Venerology Kepler University Hospital Linz Austria

**Keywords:** CD23, FcεRI, food allergy, galectin‐3, galectin‐9, IgE receptor

## Abstract

**Background:**

IgE‐mediated food allergy is the result of an aberrant immune response involving the interaction of a food allergen with its specific IgE bound to FcɛRI, the high affinity IgE receptor, on mast cells. Allergen‐specific IgE also binds to soluble binding factors, but, their expression and role in food allergy is not well characterized. Here, we assess the prevalence and relevance of soluble IgE binding factors in food allergy and tolerance.

**Methods:**

We measured serum levels of four IgE binding factors, that is, galectin‐3, galectin‐9, soluble FcɛRI (sFcεRI) and soluble CD23 (sCD23) in 67 adults sensitized to peanut or hazelnut and sFcɛRI in 29 children sensitized to hen's egg. Adults without food allergen sensitization (*n* = 17) served as healthy controls. We compared serum levels of patients and controls and assessed them, in the former, for links to clinical features including allergy and tolerance.

**Results:**

Serum levels of sFcɛRI and sCD23, but not galectin‐3 and galectin‐9, significantly differ in food‐sensitized patients as compared to healthy controls. A subgroup (28%) of peanut and hazelnut allergic patients had elevated sFcεRI levels, that were associated with higher total and specific IgE levels. Furthermore, sFcεRI levels were significantly higher in tolerant subjects compared to allergics. Among hazelnut allergic patients, those with high sFcεRI levels tolerated the highest protein amounts in the oral food challenge.

**Conclusion:**

sFcɛRI but not sCD23, galectin‐3 and galectin‐9 might play a role in the pathophysiology of food allergy. Its functional role or use as biomarker should be assessed in further studies.

## 1 INTRODUCTION

Food allergy is a frequent, serious and potential fatal allergic disease, with increasing rates over the last decades.^1^, ^2^, ^3^ It often starts in early childhood and can be transient, but also persistent into adulthood. Peanut and tree nut allergies typically persist and bear the potential to cause severe anaphylactic reactions.^4^, ^5^, ^6^ Hen's egg allergy can also lead to severe reactions. In most cases, this food allergy is transient, affecting mostly young children, with a high rate of spontaneous remission after a few years.^7^, ^8^

Food allergy is caused by specific immunoglobulin E (sIgE) bound to the high affinity receptor for IgE (FcεRI) on mast cells and basophils. Its crosslinking by food allergens leads to degranulation and the release of histamine and other proinflammatory mediators, which cause the clinical symptoms of food allergy. Allergen‐specific IgE also binds to its low affinity receptor, CD23, expressed by follicular B cells, T‐cells, monocytes, Langerhans cells, eosinophils, and macrophages.^9^ Additionally to the membrane bound receptors, soluble IgE‐binding factors exist: soluble FcεRI (sFcεRI), soluble CD23 (sCD23) as well as galectin‐3 and galectin‐9, but their role and relevance in food allergy are not well characterized.^10^, ^11^

sFcεRI is a truncated receptor consisting of the alpha chain, the IgE‐binding domain of the membrane bound FcεRI complex. In vitro, sFcεRI can block IgE binding to FcεRI on cell surfaces and inhibits basophil activation as well as anaphylaxis in mouse models.^12^, ^13^ sFcεRI has been proposed as a biomarker for atopic diseases like atopic dermatitis and allergic asthma. It has also been proposed as a biomarker to identify patients who tolerate drug desensitization protocols.^15^ Despite these extensive previous data, little is known about sFcεRI in food allergy.^14^, ^15^ The same holds true for sCD23, which results from a proteolytic cleavage of the transmembrane form, for example, this cleavage can be accomplished by an enzymatic activity of the house dust mite allergen Der p 1.^10^, ^16^, ^17^ All described isoforms of sCD23 possess the globular lectin head domain and therefore the IgE binding site. However, their different sizes seem to have different functional implications, for example, different potencies to inhibit or enhance IgE production.^18^ In addition, sCD23 has been suggested as a biomarker for atopy.^19^, ^20^, ^21^, ^22^, ^23^, ^24^, ^25^

Galectin‐3 and galectin‐9 are carbohydrate‐binding proteins and have a large number of intracellular and extracellular binding partners; for example, galectin‐3 not only interacts with IgE but also with FcεRI and therefore can activate mast cells in an IgE‐dependent and ‐independent manner.^26^, ^27^ Galectin‐3 plays a role in a variety of inflammatory processes, including neutrophil adhesion, chemoattraction of monocytes and macrophages as well as migration of dendritic cells.^28^ Due to its various interaction partners, elevated galectin‐3 serum levels can be found in many disorders including autoimmune diseases and cancer.^29^, ^30^, ^31^, ^32^, ^33^ Galectin‐9, which is also elevated in various diseases, binds to IgE stronger than galectin‐3. Galectin‐9 was shown to inhibit mast cell degranulation by depriving IgE of binding to its antigen in a concentration‐dependent manner.^34^, ^35^

Although in vitro soluble IgE binding factors interfere with IgE dependent mast cell activation their clinical implication in food allergy remains to be characterized in detail.

The humanized monoclonal anti‐IgE antibody omalizumab binds IgE molecules in the same domain as FcεRI and therefore, binds free IgE circulating in serum without triggering mast cell degranulation. Clinical trials using this monoclonal antibody in patients with food allergy have shown to be efficacious as these patients developed transient tolerance to higher amounts of allergen consumption.^36^, ^37^

Like omalizumab, the soluble IgE receptors have the ability to bind IgE and thus may function as natural protectors from allergic reactions, including food allergy, by reducing the amount of available IgE molecules to bind to membrane receptors. We, therefore, hypothesized that (1) sFcεRI, sCD23, galectin‐9, and galectin‐3 levels are increased in patients with food allergen‐specific IgE, (2) are linked to total IgE levels, (3) correlate with allergen sensitization and (4) correspond to disease activity.

To test this hypothesis, we assessed adult patients with peanut and hazelnut allergy as well as pediatric patients with hen's egg allergy for their serum levels of these IgE binding factors as well as for their total IgE levels and clinical features including tolerance.

## 2 METHODS

### 2.1 Patients

Patient material from 96 patients was obtained from three different clinical studies in the Division of Allergy and Immunology at the Department of Dermatology, Venerology and Allergology, Charité‐Universitätsmedizin Berlin CCM and the Department of Pediatric Respiratory Medicine, Immunology and Critical Care Medicine, Charité‐Universitätsmedizin Berlin Campus Virchow. All clinical studies were approved by the local ethics committee (Charité – Universitätsmedizin Berlin, EA2/143/11, EA2/033/19 and EA2/136/18). All patients or their parents gave written informed consent, before they were enrolled in the studies. We included peanut and hazelnut sensitized adults (*n* = 67) as well as hen's egg sensitized children (*n* = 29) to reflect food allergy heterogeneity. The control group consisted of 17 serum samples from non‐sensitized adults with no history of food allergy, negative skin prick test (SPT) and/or no sIgE toward peanut and hazelnut (non‐allergic). Sensitized patients, with detectable elevated sIgE levels to food allergens and/or positive SPT, were divided into two groups according the comprehensive standardized diagnostic workup: sensitized as well as allergic (hereafter allergic, *n* = 58), sensitized but tolerant patients (hereafter tolerant, *n* = 38).

Clinical characteristics and demographics of all patients and healthy controls are presented in Supplemental Tables S1 and S2. There was no significant difference in sex and age between our adult subgroups.

Peanut, hazelnut or hen's egg allergy status was diagnosed by oral food challenge (OFC), typically double‐blinded, placebo‐controlled, or ‐in few cases‐by a clear food allergy history and a positive SPT and sIgE determination. The OFCs were performed in accordance with clinical routine practice of the Charité‐Universitätsmedizin Berlin, partly based on PRACTALL international guidelines for OFCs and partly based on in‐house standard operating procedures and stopped using standardized stopping criteria based on PRACTALL guidelines.^38^ If determined, threshold levels of the OFC reflect at which amount of food intake patients started to have objective symptoms. Samples were taken at baseline.

Patients had not taken systemic steroids and H_1_‐antihistamines in the last 2 weeks and 3 days before sampling, respectively. Patient blood samples were stored at −80°C until used for this study.

### 2.2 Routine laboratory assessments

IgE levels were measured using the ImmunoCAP System^®^ (Phadia Laboratory Systems, Thermo Fisher Scientific Inc, Uppsala, Sweden) at a central laboratory (Labor Berlin GmbH, Berlin, Germany). Serum was analyzed for total IgE and IgE against the following food allergens: peanut, Ara h 1, Ara h 2, Ara h 3, Ara h 8, Ara h 9, hazelnut, Cor a 1, Cor a 8, Cor a 9, Cor a 14 and hen's egg. Total IgE levels >100 kU/l were considered as elevated, specific IgE levels of ≥0.35 kU/l as sensitization (0.00–0.34 CAP‐Class 0, 0.35–0.70 CAP‐Class 1, 0.70–3.50 CAP‐Class 2, 3.50–17.5 CAP Class 3, 17.5–50.0 CAP Class 4, 50.0–100.0 CAP‐Class 5, >100.0 CAP‐Class 6).

### 2.3 Soluble IgE binding factors

The soluble IgE receptor levels were measured using the following commercially available ELISA kits according to manufacturer's protocol: Human FcεRI ELISA Kit (BMS2101‐2TEN, Invitrogen, Thermo Fisher Scientific, Waltham, MA, USA), Human sCD23 ELISA Kit (BMS227‐2TEN, Invitrogen, Thermo Fisher Scientific, Waltham, MA, USA), Human LGALS3/Galectin‐3 ELISA Kit (RAB0661, Sigma‐Aldrich, St. Louis, MO, USA) and Human Galectin‐9 ELISA (RAB1475, Sigma‐Aldrich, St. Louis, MO, USA). Soluble FcεRI was measured in adults as well as in children, whereas sCD23, galectin‐3 and galectin‐9 were not measured in children. For sFcεRI both total and IgE bound levels were measured in 33 patients. As levels did not differ and were tightly correlated, for remaining measurements only total levels were determined (see Supplement 3). sFcεRI, galectin‐3 and galectin‐9 levels were given in ng/ml and sCD23 levels were given in U/ml. Patients' serum samples were measured in duplicates. The optical density was read at 450 nm using the Victor V reader (Perkin Elmer, Waltham, MA, USA).

### 2.4 Statistical analyses

Statistical analyses were performed using GraphPad Prism Version 9.0. Descriptive statistic values are given as median and interquartile range (median [IQR]). Binominal variables were analyzed using Chi‐square test or Fisher Exact test for small categorical numbers (<5). Non‐parametric continuous variables were compared using Man‐Whitney‐U test. Correlations were analyzed using spearman rank test. *p* < 0.05 was considered to indicate statistical significance.

## 3 RESULTS

### 3.1 Levels of sFcεRI and sCD23, but not galectin‐3 and galectin‐9 differ between food allergen‐sensitized subjects and healthy controls

**FIGURE 1:**
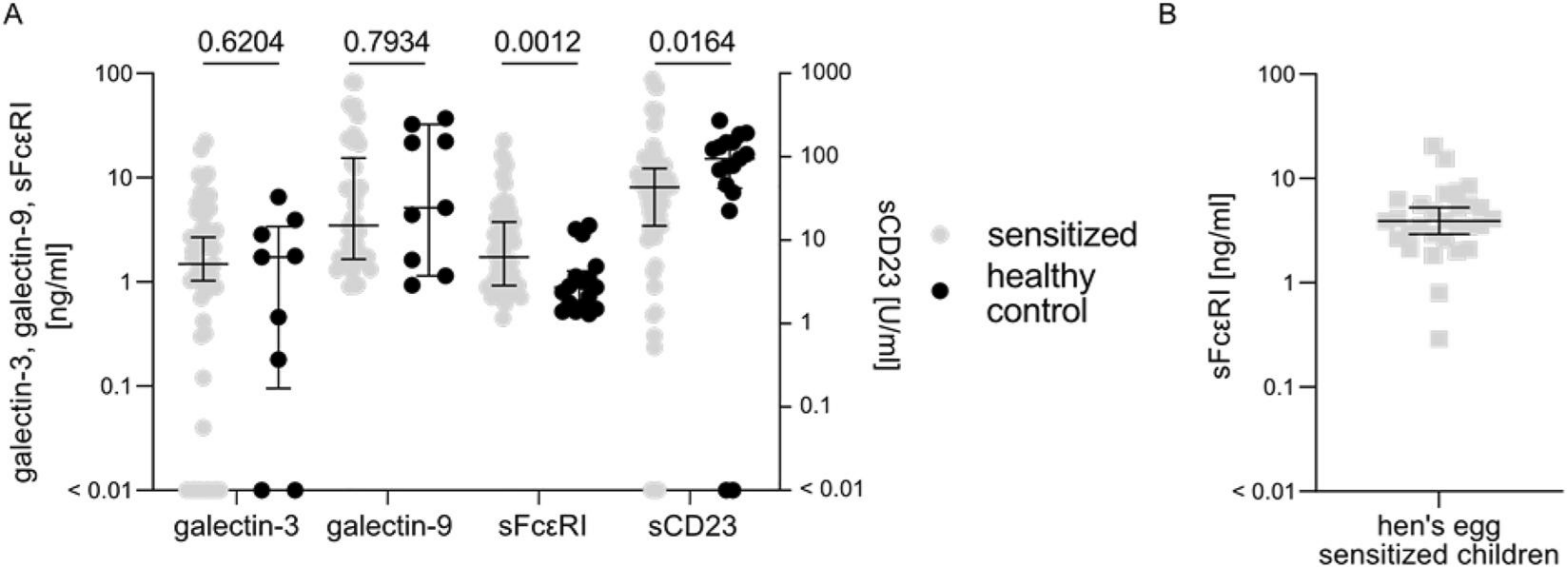
Levels of soluble high and low affinity IgE receptors are up and downregulated, respectively, in food allergen‐sensitized subjects. (A) Detection of soluble IgE receptor levels by ELISA in peanut and hazelnut sensitized adults (gray dots) and healthy controls (black dots). Galectin‐3 levels were detected in 55 sensitized patients and 9 healthy controls. Galectin‐9 was measured in 50 sensitized patients and 9 healthy controls. Soluble FcεRI and sCD23 levels were investigated in 63 and 66 sensitized patients, respectively, and 17 healthy controlss. Galectin‐3, Galectin‐9 and sFcεRI levels were measured in ng/ml (left ordinate) and sCD23 levels were measured in U/ml (right ordinate). Undetectable Galectin‐3 and sCD23 levels are depicted as levels <0.01. Mann‐Whitney test was used for group comparison and *p*‐values <0.05 were considered significant. (B) Detection of sFcεRI levels in 29 hen's egg sensitized children (gray squares).

Adult patients sensitized (allergic or tolerant) to peanut and/or hazelnut had similar serum levels of galectin‐3 and galectin‐9 as compared to non‐sensitized healthy controls (Figure 1; for patient and healthy control demographics see Supplemental Table S1). In contrast, sFcεRI serum levels in sensitized patients were significantly higher (median [IQR]: 1.73 [2.83] ng/ml) than those of healthy controls (0.89 [0.71] ng/ml, *p* = 0.0012). In peanut and tree nut sensitized patients, sFcεRI serum levels varied considerably, from 0.45 to 22.46 ng/ml. Using the 90% percentile from the measured healthy control sera, 19 sensitized patients (=28%) showed elevated sFcεRI serum levels (levels greater than 3.26 ng/ml). Taking the previously proposed cut‐off value for clinically relevant sFcɛRI levels (>2 ng/ml), 30 (=48%) sensitized patients presented elevated levels.^14^, ^15^

In addition, allergen sensitized patients exhibited significantly lower sCD23 levels compared to non‐sensitized healthy controls, that is, 43.03 [57.95] U/ml versus 94.68 [107.39] U/ml, *p* = 0.0246, Figure 1A. Peanut and hazelnut sensitized patients exhibited sCD23 levels ranging from undetectable to 847.4 U/ml (Figure 1A). Since several healthy controls were also having undetectable sCD23 levels, we used the 25% percentile from healthy controls as cut‐off value (41.81 U/ml). Thirty‐two sensitized patients (=48%) showed sCD23 serum levels lower than 41.81 U/ml.

In hen's egg sensitized pediatric patients (*n* = 29; for patient demographics see Supplemental Table S2), sFcεRI levels were ranging from 0.29 to 15.45 ng/ml, also presenting elevated median sFcεRI levels, that is, 3.47 [0.88] ng/ml (Figure 1B).

### 3.2 Soluble FcεRI but not sCD23 levels are associated with total IgE levels in food allergen‐sensitized patients

**FIGURE 2:**
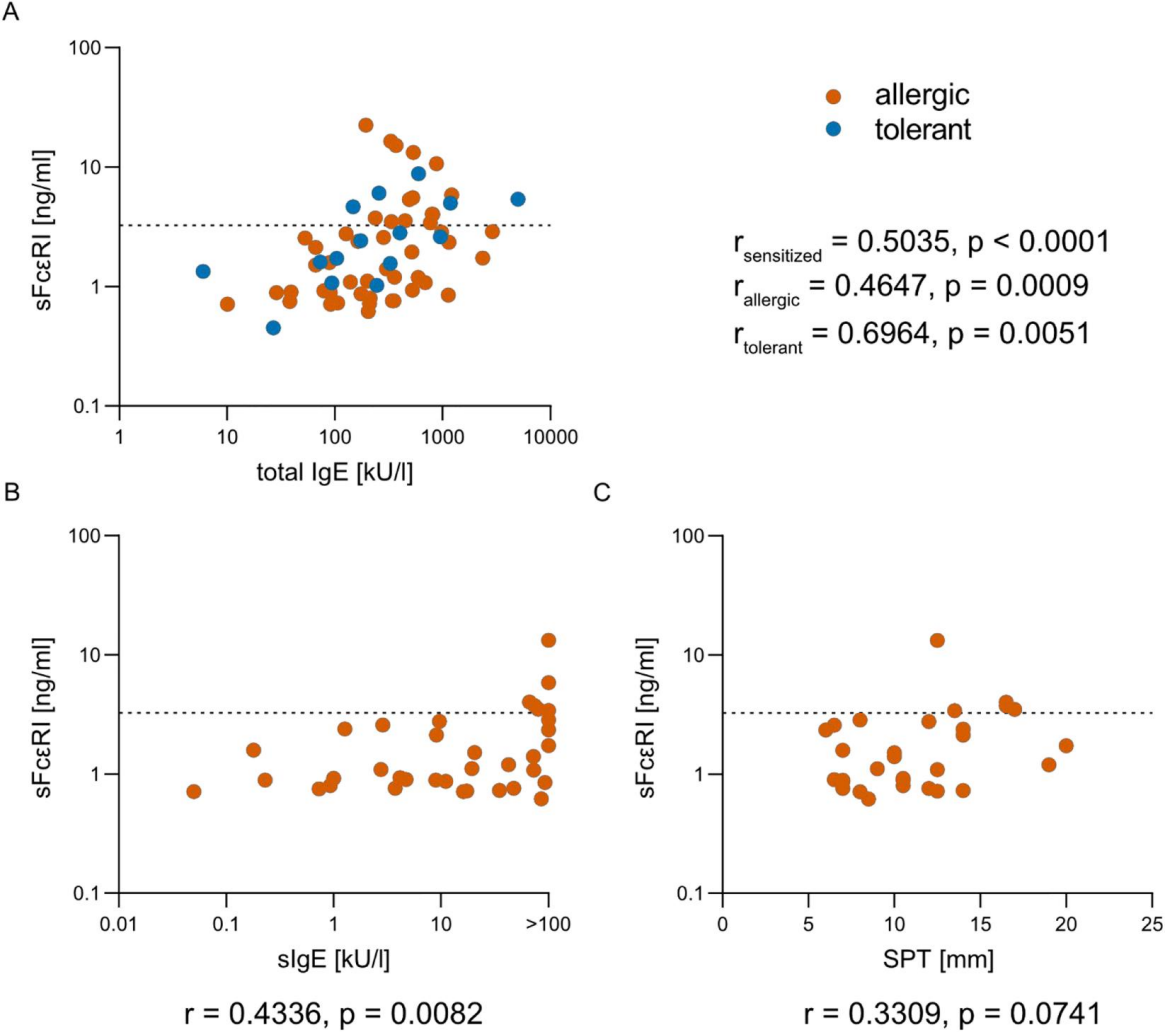
Soluble FcεRI serum levels are associated with total IgE levels and peanut allergen sensitization. (A) Correlation between sFcεRI and total IgE in peanut and/or hazelnut sensitized patients. Patient cohort is subdivided in allergic patients (orange, *n* = 48) and sensitized but tolerant subjects (blue, *n* = 15). (B) sFcεRI levels and sIgE against peanut positively correlate in peanut allergic patients (*n* = 36). (C) Correlation between sFcεRI serum levels and wheal size after SPT in peanut allergic patients (*n* = 30). (A/B/C) Spearman's correlation coefficient was used to measure the strength and direction of association. The dotted line represents the 90% percentile of healthy controls.

Peanut and/or hazelnut sensitized patients with elevated sFcεRI levels showed higher total IgE levels compared to patients without elevated sFcεRI levels (529.0 [479.0] ng/ml vs. 188.0 [309.9] ng/ml, *p* = 0.0005), and sFcεRI was positively correlated with total IgE levels (*r* = 0.50, *p* < 0.0001, Figure 2A). In contrast, serum sIgE levels against peanut and hazelnut were similar in sensitized patients with and without elevated sFcɛRI (Peanut: 66.6 [98.8] ng/ml vs. 6.9 [39.6] ng/ml, *p* = 0.2811; hazelnut: 12.4 [19.0] ng/ml vs. 9.0 [24.6] ng/ml, *p* = 0.5635).

Nineteen out of 29 (=66%) children sensitized to hen's egg showed sFcεRI levels greater than 3.26 ng/ml.

In contrast to sFcεRI, low sCD23 levels were not associated with any of the assessed features (Supplement 4).

### 3.3 Soluble FcεRI are linked to total IgE levels as well as allergen testing in food allergy

Fourteen of the 19 (= 74%) peanut and hazelnut sensitized adults with elevated sFcεRI levels were allergic toward their culprit allergen. Therefore, we also investigated the link between total IgE levels as well as allergen sensitization in peanut and hazelnut allergic patients with normal and elevated sFcεRI levels (Table 1). Allergic patients with elevated sFcεRI levels had significantly higher total IgE levels compared to patients with low levels (511 [469] kU/l vs. 203 [433] kU/l, *p* = 0.0061). Also in peanut and hazelnut allergic patients (depicted in orange), sFcεRI positively correlate with total IgE levels (*r* = 0.4647, *p* = 0.0009, Figure 2A).

**TABLE 1:** Characteristics of nut allergic patients with normal and elevated (>3.26 ng/ml) sFcεRI levels.

	Normal sFcεRI (*n* = 34)	Elevated sFcεRI (*n* = 14)	*p*‐Value
Total IgE (kU/l)	203 ± 433	511 ± 469	**0.0061**
sIgE peanut extract	10.4 ± 51.4	90.1 ± 27.7	**0.0019**
sIgE hazelnut extract	34.3 ± 49.7	15 ± 19.5	0.2284
Pollen‐associated food allergy (*n*)	2 (6%)	5 (36%)	**0.0167**
SPT peanut (mm)	10 ± 5.5	16.5 ± 3.8	**0.0080**
SPT hazelnut (mm)	8.5 ± 8.5	5.75 ± 0.9	0.4309

*Note*: Data was given as median + IQR for total IgE, sIgE, SPT, and as number (%) for pollen‐associated food allergy. Mann Whitney test was used for total IgE, sIgE, SPT and Chi‐square‐test for pollen‐associated food allergy.

*p*‐values <0.05 were considered as statistically significant. Significant values are displayed in bold.

Unlike in peanut‐sensitized subjects, elevated sFcεRI serum levels in peanut allergic patients were associated with higher sIgE levels and SPT reactions toward peanuts. Along, sIgE against peanut positively correlate with sFcεRI levels (*r* = 0.4336, *p* = 0.0082, Figure 2B) in peanut allergic patients. However, the positive correlation between wheal size after SPT and sFcεRI levels in peanut allergic patients did not reach statistical significance (*r* = 0.3309, *p* = 0.0741, Figure 2C).

Such differences were not seen in regard to hazelnut. Furthermore, nut allergic patients with elevated sFcεRI levels had more frequently documented diagnosed secondary pollen‐associated food allergies (no sensitization against storage proteins: Ara h 1, Ara h 2, Ara h 3, Ara h 6 or Cor a 9, Cor a 14).

Six out of eight (=75%) children with hen's egg allergy showed elevated sFcεRI levels. However, due to low numbers no link between total IgE, sIgE and SPT was investigated.

### 3.4 Tolerance to sensitized food is associated with higher sFcεRI levels

**FIGURE 3:**
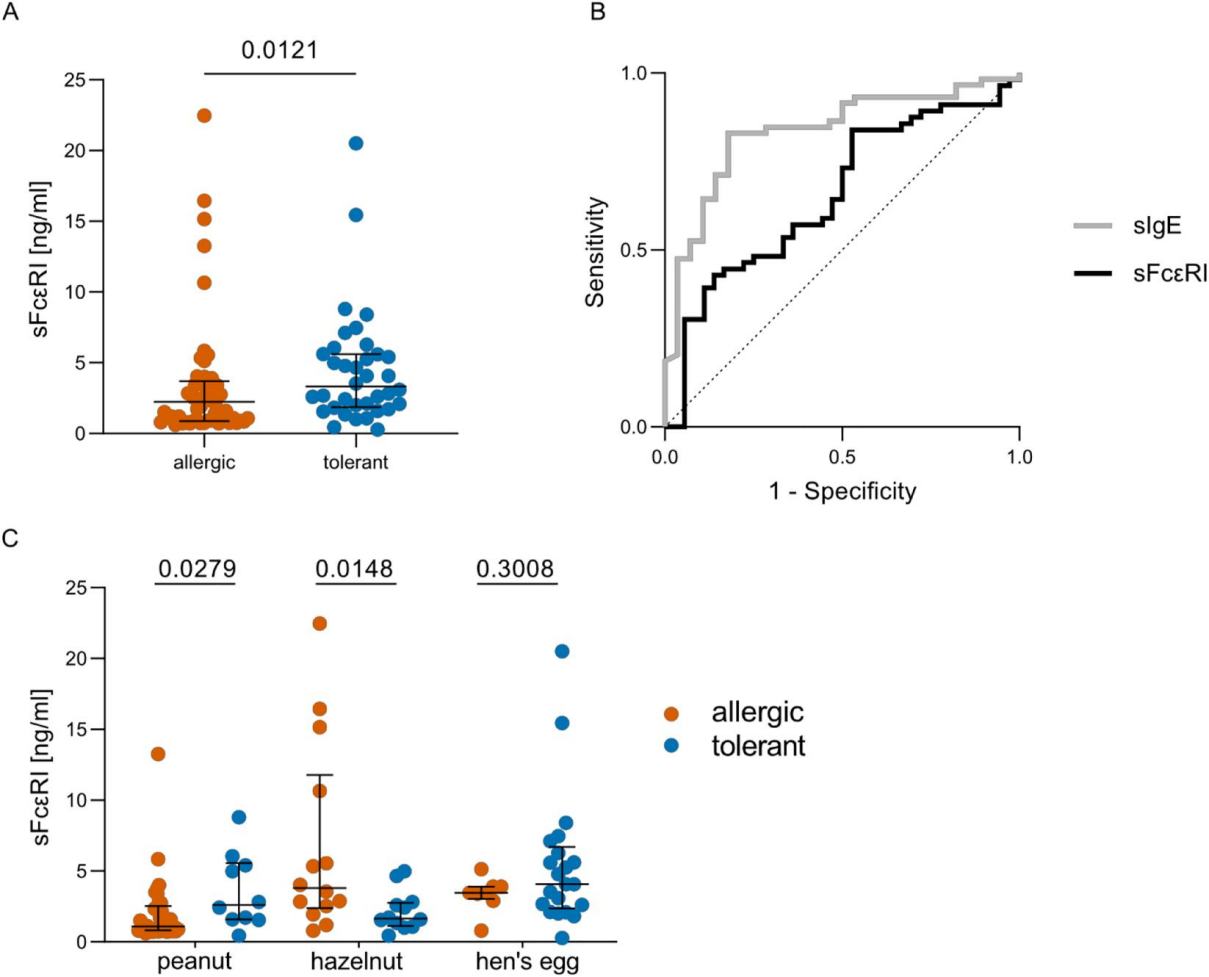
Soluble FcεRI serum levels as predictor of tolerance and allergy. (A) Bar Chart shows individual sFcεRI serum levels of tolerant (blue) and allergic (orange) individuals with median and IQR. Mann‐Whitney test was performed and *p*‐values < 0.05 were considered significant. (B) Receiver operator characteristics shows the ability of sIgE (gray, AUC = 0.8357) and sFcεRI (black, AUC = 0.6548) to discriminate between sensitization and allergy. (C) Individual sFcεRI serum levels of tolerant (blue) and allergic (orange) individuals according to the allergens (peanut, hazelnut and hen's egg) with median and IQR. Mann‐Whitney test was performed and *p*‐values < 0.05 were considered significant.

In total, 38 individuals of the 96 peanut, hazelnut and hen's egg sensitized subjects (= 40%) had a documented tolerance toward their sensitized food allergen. Significantly higher sFcεRI levels can be detected in tolerant compared to allergic patients (3.3 [3.7] ng/ml vs. 2.4 [2.8] ng/ml, *p* = 0.0121, Figure 3A). However, as shown in Figure 3B, sIgE levels were better predictors for food allergy in our small cohort than sFcεRI levels.

Within the different cohorts, 10 patients (= 22%) with peanut sensitization were tolerant upon ingestion, 12 patients (= 46%) with hazelnut sensitization and 21 patients (= 72%) with hen's egg sensitization.

In peanut tolerant patients sFcεRI serum titers were slightly, but significantly higher than in allergic ones (2.62 [3.98] ng/ml vs. 1.10 [1.72] ng/ml, *p* = 0.0279). A similar trend, but without significance, was seen in sensitized but tolerant children to hen's egg (4.08 [4.34] ng/ml vs. 3.47 [0.88] ng/ml, *p* = 0.3008), possibly due to the small allergic cohort.

No such correlation was seen in hazelnut sensitized patients: sFcεRI serum levels were markedly and significantly higher in hazelnut allergic patients than in tolerant ones (3.80 [9.39] ng/ml vs. 1.66 [1.63] ng/ml, *p* = 0.0148).

For several peanut and hazelnut allergic patients, clinical parameters regarding OFC threshold levels were available. We observed high levels of sFcɛRI (>10 ng/ml) in three out of four hazelnut allergic patients who reacted at the highest allergen dose during OFC (1.8 or 2.1 g hazelnut‐protein, data not shown). In none of the 8 remaining hazelnut allergic patients that reacted at lower allergen concentrations, such high levels were detected. For the group of peanut allergic patients, the threshold concentration of the allergen in the OFC was not documented for patients with the highest sFcεRI levels.

Measurement of sCD23, galectin‐3 or galalectin‐9 did not result in any differences among peanut and hazelnut tolerant or allergic patients in our nut allergic patient cohort (Supplement 5).

## 4 DISCUSSION

Analyzing soluble IgE binding factors in food allergen sensitized patients and non‐allergic controls, we showed that sCD23 serum levels are significantly lower and sFcεRI serum levels are significantly elevated in food sensitized individuals, while there is no difference in galectin‐3 and galectin‐9 levels.

sCD23 levels in food allergen sensitized patients were considerably varying from undetectable to 847.4 U/ml, with about half of the patients showing low levels (often undetectable levels) compared to healthy controls. In literature, the role of sCD23 in atopic diseases remains unclear. There are reports showing increased sCD23 serum levels of allergic rhinitis patients monosensitized to grass pollen during the symptomatic period and higher sCD23 levels in asthmatic patients compared to healthy controls.^19^, ^39^ However, there are also papers showing negative associations between sCD23 levels and atopy.^40^, ^41^ One potential explanation could be different isoforms having different functional implications, for example, monomers inhibit IgE synthesis while tetramers upregulate IgE synthesis.^18^ This could also affect antigen‐specific IgE synthesis.

Previously Moñino‐Romero et al. presented sFcεRI as biomarker for IgE‐mediated diseases. Atopic patients (patients with food allergy, insect venom allergy, atopic asthma or atopic dermatitis) showed significantly higher sFcεRI levels compared to controls and levels were associated with total IgE. However, they could find no differences between food allergic and sensitized individuals as well as correlations with sIgE levels.^14^ In line with these findings, we have shown that compared to healthy controls sFcεRI was elevated in about one third of the peanut and hazelnut allergic subjects and high levels correlate with total IgE in both adult cohorts. Median sFcεRI levels were also high in hen's egg sensitized children, where two‐third of the patients exhibited sFcεRI levels greater than 3.26 ng/ml. In contrast, we found higher sFcεRI levels in tolerant subjects than allergic ones and an association between sFcεRI and specific allergen sensitization (sIgE and SPT) in peanut allergic patients.

A potential functional role of IgE‐binding factors in food allergy, has already been proposed for histamine releasing factor (HRF).^42^ In the context of the pathogenesis of late‐phase allergic reactions MacDonald et al. reported on the influence of IgE heterogeneity (IgE+: IgE that interacts with HRF and IgE−: IgE that does not interact with HRF) on clinical disease severity of atopic patients.^43^ They conclude that the release of histamine by basophils during late‐phase reactions is IgE‐dependent and relies on the interaction of IgE and histamine‐releasing factor. The interaction of soluble IgE receptors with IgE might be an explanation for the different binding capacities of histamine‐releasing factor to varying IgE types and its influence on disease severity.

Till today, OFC, even though being time‐consuming and potentially harmful for the patient, is still the gold standard for food allergy diagnosis.^44^ Further routine diagnostic tools include SPT and sIgE measurements, which allow assessment of the sensitization status, but not the true clinical reactivity of the patient toward the culprit allergen.^45^, ^46^ Additional tests like the basophil activation test have been developed, but these tests require fresh blood sampling and are partly challenging. Accordingly, the need for an easy assessable lab marker would be welcomed. Assessment of sFcεRI could be easily done, even from stored frozen samples.

Our data showed that sFcεRI serum levels, but none of the other IgE binding factors, significantly differed between tolerant and allergic peanut sensitized patients, but not hazelnut sensitized patients. In a report on tolerance induction in platin sensitized patients^15^ elevated sFcεRI serum levels were associated with successful tolerance induction. However, for our patient cohort the diagnostic ability to discriminate between tolerance and food allergy was higher for sIgE levels than sFcεRI.

Besides, our data raises the question why sFcεRI levels behave differently between patients allergic to different foods. There are two main possibilities: (i) Soluble FcεRI might rather mirror the patient's atopy status than being specific for food allergy. In solely food allergic patients, even low sIgE against for example, peanut can already lead to severe reactions, while for example, hazelnut allergy is often pollen‐allergy associated with many sensitizations and comorbidities like rhinitis and asthma. Low sFcεRI levels could therefore indicate general low atopy including a lower overall allergy risk. (ii) Soluble FcεRI levels might change over time and different food allergies have a different potential for resolution. Accordingly, high levels in patients might indicate ongoing desensitization mechanisms in atopic patients.

The overall higher levels seen in the more often pollen‐allergy associated hazelnut‐allergic patient cohort would be in line with the first hypothesis, whereas the significantly higher sFcεRI levels in tolerant subjects indicate a possible pathophysiological relevance according to hypothesis two. In such sensitized but tolerant patients sFcεRI would bind allergen‐sIgE and therefore prevents mast cell activation and would explain why hazelnut‐allergic patients with high sFcεRI levels tolerate the highest amount of allergen during the OFC. Unfortunately, in peanut‐allergic patients OFC data of the patients with highest sFcεRI levels were missing and no additional information can be drawn for the role of sFcεRI in this patient cohort.

As third pathophysiological possibility, also, both mechanisms could be present in food allergic patients depending on the atopic background and the status of the allergy. Given this circumstance, the use of sFcεRI as a stand‐alone biomarker might be limited, but could, maybe together with other factors, give a clearer picture of the allergic status of a patient and might be of use as a follow up marker during tolerance induction, for example, with the newly developed oral immunotherapy for peanut sensitized children.^47^

This project was set out as an exploratory study with low patient numbers and a retrospective cross‐sectional single center study design.

For ethical reasons no age‐matched, non‐allergic controls exist for the hen's egg sensitized children and sCD23, galectin‐3 and galectin‐9 were not measured in this cohort, due to limited amount of serum sample. However, to our knowledge this is the first study indicating a potential relevance of soluble IgE binding factors, especially sFcεRI in food allergy.

As the above mentioned limitations could potentially have influenced the study outcome a prospective long‐term follow up study with a larger cohort is needed to further clarify the relevance of sFcεRI in nut and other food allergies and its precise role in the pathophysiology.

## AUTHOR CONTRIBUTIONS

**Sabine Altrichter**: Conceptualization (lead); Funding Acquisition (equal); Methodology (lead); Project Administration (lead); Resources (equal); Supervision (equal); Validation (equal); Writing – Original Draft Preparation (equal). **Kirsten Beyer**: Conceptualization (supporting); Funding Acquisition (equal); Resources (equal); Writing Review & Editing (supporting). **Monique Butze**: Formal Analysis (supporting); Investigation (supporting); Writing Review & Editing (supporting). **Josefine Dobbertin‐Welsch**: Investigation (supporting); Resources (equal); Writing Review & Editing (supporting). **Sabine Dölle‐Bierke**: Conceptualization (supporting); Funding Acquisition (equal); Investigation (supporting); Resources (equal); Writing Review & Editing (supporting). **Marcus Maurer**: Funding Acquisition (equal); Resources (equal); Supervision (supporting); Writing Review & Editing (supporting). **Sherezade Moñino‐Romero**: Conceptualization (supporting); Formal Analysis (supporting); Methodology (supporting); Writing Review & Editing (supporting). **Jörg Scheffel**: Methodology (supporting); Supervision (equal); Writing Review & Editing (supporting). **Carolin Steinert**: Data Curation (lead); Formal Analysis (lead); Investigation (lead); Methodology (supporting); Validation (equal); Visualization (lead); Writing – Original Draft Preparation (equal).

## CONFLICT OF INTEREST STATEMENT

Carolin Steinert: No conflict of interests. Sherezade Moñino‐Romero: No conflict of interests. Monique Butze: No conflict of interests. Jörg Scheffel: No conflict of interests in relation to this paper. Outside of it JS has conducted studies for, received research funds/was advisor for Allakos, Ascilion, AstraZeneca, CSL Behring, Escient, Novartis, Sanofi, Third Harmonic Bio, ThirdRock, ThermoFisher. Sabine Dölle‐Bierke: No conflict of interests. Josefine Dobbertin‐Welsch: No conflict of interests. Kirsten Beyer: No conflict of interests in relation to this paper. Outside of it, KB received personal fee from Aimmune, Bencard, Danone, DBV, Hipp, Hycor, Jenapharma, Infectopharm, Mylan/Meda, Nestle, Novartis and ThermoFisher. Marcus Maurer: No conflict of interest in relation to this paper. Outside of it, MM is or recently was a speaker and/or advisor for and/or has received research funding from Allakos, Amgen, Aralez, ArgenX, AstraZeneca, Celldex, Centogene, CSL Behring, FAES, Genentech, GIInnovation, GSK, Innate Pharma, Kyowa Kirin, Leo Pharma, Lilly, Menarini, Moxie, Novartis, Pfizer, Roche, Sanofi/Regeneron, Third Harmonic Bio, UCB, and Uriach. Sabine Altrichter: No conflict of interest in relation to this paper. Outside of it, SA has conducted studies for/was advisor for/was speaker for AstraZeneca, Allakos, ALK, CSLBehring, LeoPharma, Moxie, Novartis, Sanofi, Takeda, Thermofisher.

## Supporting information

Supporting Information S1
