# Supplementary material for: Soluble IgE‐binding factors in the serum of food‐allergic patients: Possible pathophysiological role of soluble FcεRI as protective factor
Source: Clin Transl Allergy. 2023 Feb 7;13(2):e12222. doi: 10.1002/clt2.12222 (PMC9904993; doi:10.1002/clt2.12222)
Supplement: Supplementary file 1 — Supporting Information S1 [file CLT2-13-e12222-s001.docx]

**Supplementary tables and figures**

***Supplement 1***

|  | **Healthy Control**  **(n=17)** | **Tolerant**  **(n=17)** | **Allergic**  **(n=50)** | **P-value** |
| --- | --- | --- | --- | --- |
| **Age [years]** | 32 (6.5) | 32 (22) | 27 (10.3) | 0.0983 |
| **Female, n (%)** | 12 (70.6) | 11 (64.7) | 33 (66) | 0.8986 |
| **Total IgE [kU/l]** | 20 (102.2)  (n = 9) | 186 (417) | 291.5 (448) | **0.0032** |
| **sIgE [kU/l]**  **(Peanut, f13)** |  | 0.87 (0.7)  (n = 10) | 18.35 (72.2)  (n = 36) | **0.0001** |
| **sIgE [kU/l]**  **(Hazelnut, f17)** |  | 9.96 (13.87)  (n = 12) | 18.8 (28.75)  (n = 14) | 0.0757 |
| **SPT [mm]**  **(Peanut extract)** |  | 4.5 (3.88)  (n = 12) | 10.5 (6.5)  (n = 33) | **0.0002** |
| **SPT [mm]**  **(Hazelnut native)** |  | 5.5 (3)  (n = 15) | 6 (6.13)  (n = 14) | 0.0502 |
| **sFcεRI [ng/ml]** | 0.89 (0.71) | 2.4 (3.64)  (n = 15) | 1.66 (2.65)  (n = 48) | **0.0042** |
| **sCD23 [U/ml]** | 94.68 (107.4) | 44.58 (51.26) | 35.52 (62.94)  (n = 49) | 0.9475 |
| **Galectin-3 [ng/ml]** | 1.73 (3.3)  (n = 9) | 1.05 (2.73)  (n = 15) | 1.83 (4.1)  (n = 40) | 0.1984 |
| **Galectin-9 [ng/ml]** | 5.15 (26.1)  (n = 9) | 3.55 (22.1)  (n = 14) | 3.35 (11.8)  (n = 36) | 0.7541 |

**Supplement 1: Patient Demographics of allergic and tolerant subjects sensitized to peanut and/or hazelnut as well as healthy controls.** In this study 17 healthy, 17 sensitized but tolerant and 50 allergic adults were assessed. Data is given as median (IQR) for age, total IgE, sIgE, SPT, sFcεRI, sCD23, galectin-3 and galectin-9 and n(%) for sex. Kruskal-Wallis test was used for age, total IgE sFcεRI, galectin-9; Mann-Whitney test for sIgE (Peanut, f13), sIgE (Hazelnut, f17), SPT (Peanut), SPT (Hazelnut) and One-way ANOVA for sCD23, galectin-3 and Chi-square test for sex with p – values <0.05 were considered significant (bold).

***Supplement 2***

|  | **Allergic**  **(n=8)** | **Tolerant**  **(n=21)** | **P-value** |
| --- | --- | --- | --- |
| **Age [years]** | 5 (7.25) | 5 (5.75)  (n = 16) | 0.6398 |
| **Female, n (%)** | 2 (25%) | 7 (33%) | >0.9999 |
| **Total IgE [kU/l]** | 664.0 (1144) | 633.5 (990)  (n = 14) | 0.5252 |
| **sIgE [kU/l]**  **(Hen‘s egg)** | 7.7 (19.7) | 1.0 (1.7)  (n = 12) | **0.0022** |
| **sFcεRI [ng/ml]** | 3.47 (0.9) | 4.08 (4.3) | 0.3008 |

**Supplement 2: Patient Demographics of hen’s egg allergic and tolerant children.** 8 allergic children and 21 sensitized but tolerant children were assessed. Data is given as median (IQR) for age, total IgE, sIgE and sFcεRI, as well as n (%) for sex. Mann-Whitney test for age, total IgE, sIgE and sFcεRI and Chi-square test for sex with p-values <0.05 were considered significant (bold).

***Supplement 3***

***
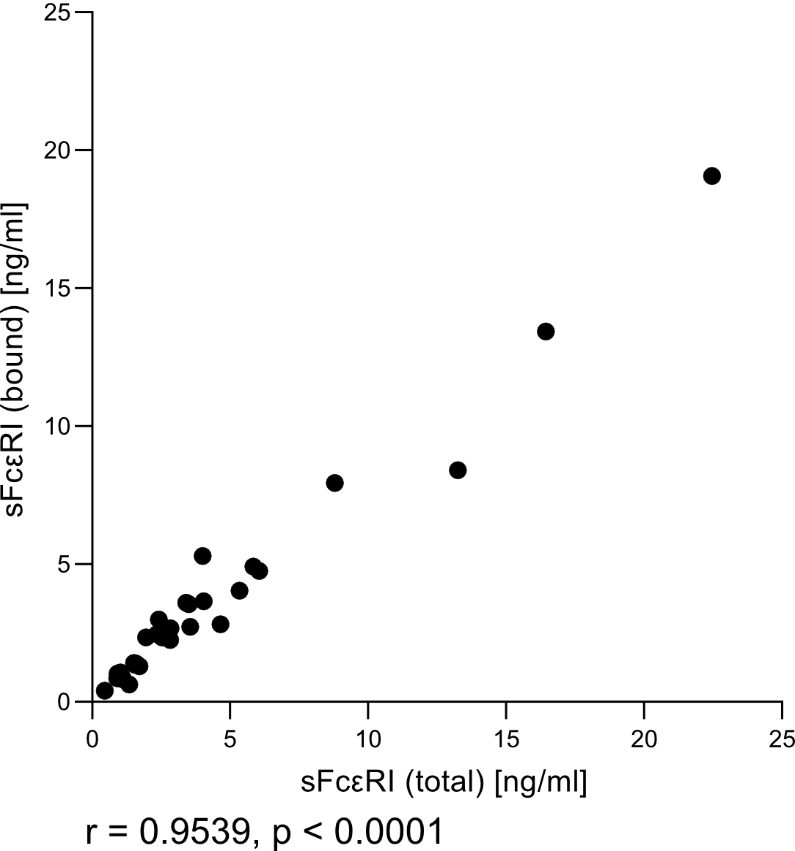
***

**Supplement 3: Total sFcεRI significantly correlates with IgE-bound sFcεRI.** Total and IgE-bound sFcεRI levels of 33 patients (r = 0.9539, p < 0.0001). Correlation was determined using Spearman‘s correlation coefficient.

***Supplement 4***

|  | **normal sCD23**  **(n = 34)** | **low sCD23**  **(n = 32)** | **P-value** |
| --- | --- | --- | --- |
| **total IgE [kU/l]** | 472.3 ± 452 | 514.7 ± 483 | 0.9164 |
| **sIgE peanut extract** | 4.16 ± 47.3  (n = 27) | 12.2 ± 77.6  (n = 21) | 0.1697 |
| **sIgE hazelnut extract** | 5.8 ± 12.3 (n = 15) | 14.6 ± 25.4  (n = 17) | 0.3699 |
| **SPT peanut [mm]** | 7.8 ± 7.6 (n = 24) | 10.3 ± 8.4 (n = 26) | 0.6332 |
| **SPT hazelnut [mm]** | 5.5 ± 4.5 (n = 27) | 4.5 ± 3.0 (n = 30) | 0.8264 |

**Supplement 4: Characteristics of peanut and hazelnut sensitized patients with normal and low (< 41.81 U/ml) sCD23 levels.** Data was given as median + IQR and Mann Whitney test was used to test for differences. P-values < 0.05 were considered as statistically significant.

***Supplement 5
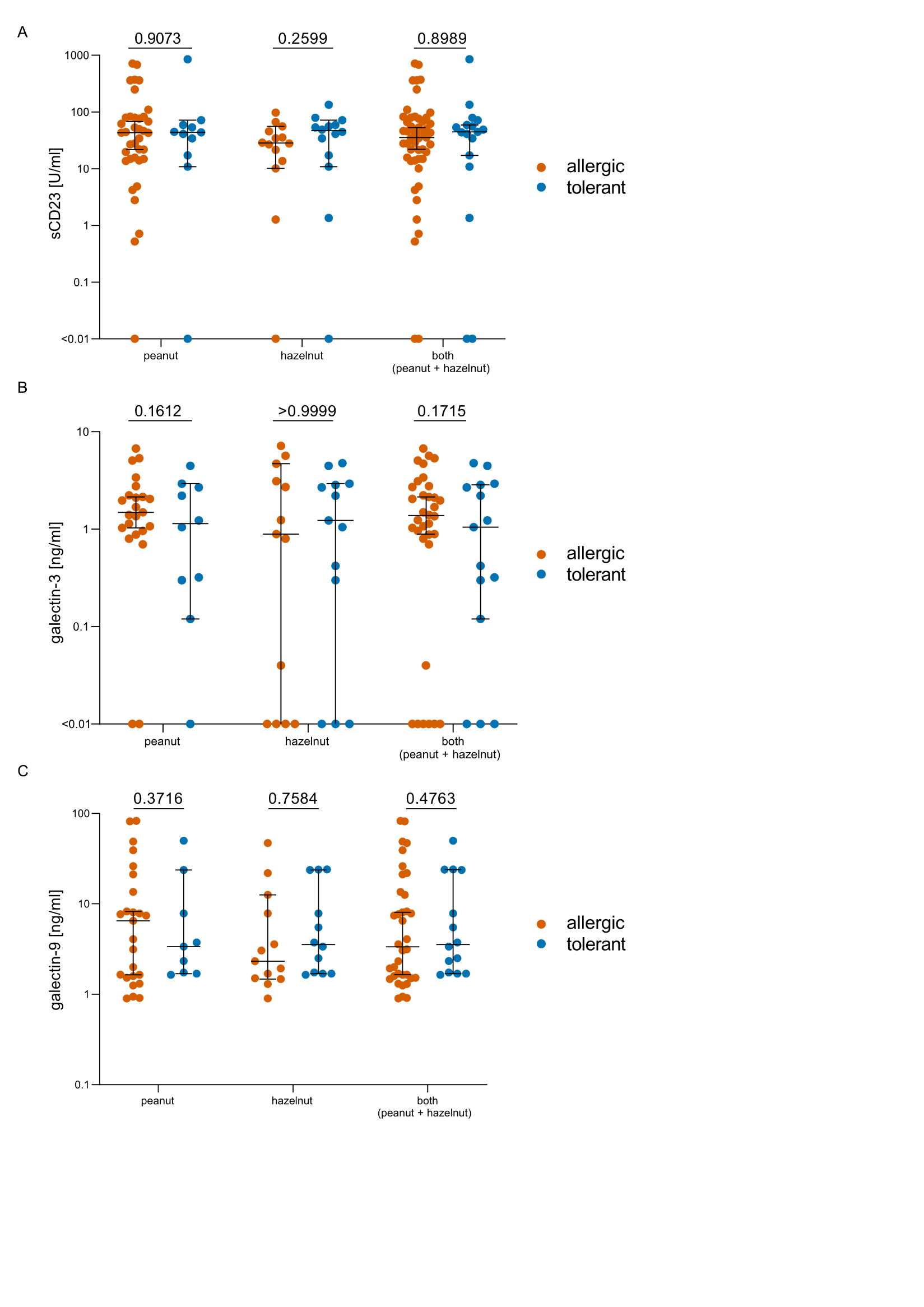
***

**Supplement 5: Galectin-3, Galectin-9 and sCD23 do not differ between tolerant and allergic patients.** A) Bar Chart shows individual sCD23 serum levels of tolerant (blue) and allergic (orange) individuals for peanut and hazelnut with median and IQR. B) Bar Chart shows individual galectin-3 serum levels of tolerant (blue) and allergic (orange) individuals for peanut and hazelnut with median and IQR.C) Bar Chart shows individual galectin-9 serum levels of tolerant (blue) and allergic (orange) individuals for peanut and hazelnut with median and IQR. A-C) Mann-Whitney test was performed and a p - value < 0.05 was considered significant.
